# Mammalian middle ear mechanics: A review

**DOI:** 10.3389/fbioe.2022.983510

**Published:** 2022-10-10

**Authors:** Maialen Ugarteburu, Robert H. Withnell, Luis Cardoso, Alessandra Carriero, Claus-Peter Richter

**Affiliations:** ^1^ Department of Biomedical Engineering, The City College of New York, New York, NY, United States; ^2^ Department of Speech, Language and Hearing Sciences, Indiana University, Bloomington, IN, United States; ^3^ Department of Otolaryngology, Northwestern University Feinberg School of Medicine, Chicago, IL, United States; ^4^ Department of Biomedical Engineering, Northwestern University, Chicago, IL, United States; ^5^ Department of Communication Sciences and Disorders, Northwestern University, Chicago, IL, United States; ^6^ The Hugh Knowles Center, Northwestern University, Chicago, IL, United States

**Keywords:** middle ear, eardrum, ossicles, ligaments, muscles, synovial joints, kinematics, mechanics

## Abstract

The middle ear is part of the ear in all terrestrial vertebrates. It provides an interface between two media, air and fluid. How does it work? In mammals, the middle ear is traditionally described as increasing gain due to Helmholtz’s hydraulic analogy and the lever action of the malleus-incus complex: in effect, an impedance transformer. The conical shape of the eardrum and a frequency-dependent synovial joint function for the ossicles suggest a greater complexity of function than the traditional view. Here we review acoustico-mechanical measurements of middle ear function and the development of middle ear models based on these measurements. We observe that an impedance-matching mechanism (reducing reflection) rather than an impedance transformer (providing gain) best explains experimental findings. We conclude by considering some outstanding questions about middle ear function, recognizing that we are still learning how the middle ear works.

## 1 Introduction

**FIGURE 1:**
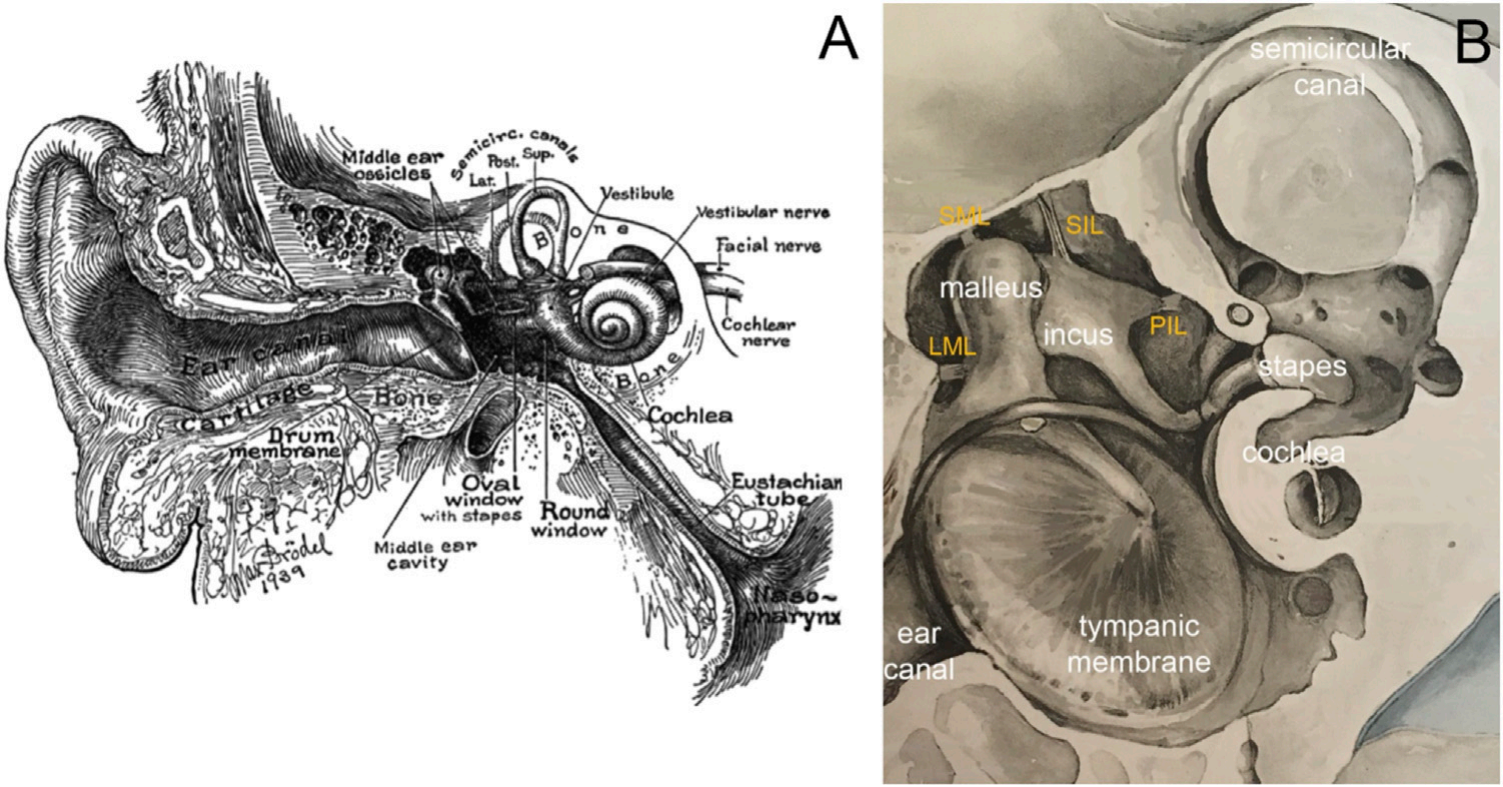
**(A)** Image of the ear in cross-section by Max Brödel. The outer ear consists of the pinna and the ear canal, the middle ear of the tympanic membrane, the middle ear cavity, ossicles, and various ligaments and muscles. The three middle ear ossicles transmit the acoustic energy to the cochlea, responsible for hearing. Nerve fibers connect the inner ear *via* the eighth cranial nerve to the brainstem. The eustachian tube ventilates the middle ear. **(B)** Modified image by Bertolini and Leutert (1982), showing a magnified view of the middle ear. Essential to holding the chain of ossicles in place are the ligaments. The image shows the lateral mallear ligament (LML), superior mallear ligament (SML), superior incudal ligament (SIL), and the posterior incudal ligament (PIL).

The ear has evolved to what might be described as an impressive example of mechanical engineering, transmitting air-borne sound *via* impedance-matching to the fluid-filled sensory organ, the cochlea (Figure 1). In mammals, a concave conical-shaped tympanic membrane or eardrum and three middle ear bones contribute to a wider bandwidth of hearing than other vertebrates. Our understanding of how sound transmission and impedance-matching by the middle ear works is informed by initial studies of anatomy in the 19th century, experimental studies of mechanical function over the past 100 years, and modeling studies that attempt to interpret the experimental findings. This paper reviews what we currently know about the mechanical function of the middle ear. We begin by considering the anatomy of the middle ear, then briefly address the effect of development on middle ear structure before introducing the basis for quantifying sound transmission through the middle ear. We are then in a position to present the focus of this review, the mechanics of the middle ear in terms of experimental results and modeling. We conclude by considering some unsolved questions on middle ear mechanics.

## 2 Anatomical aspects of the middle ear

Before considering the focus of this paper, the mechanics of the middle ear, a brief review of anatomy will provide a basis for understanding the various structures of the middle ear that contribute to sound transmission. The entrance to the middle ear is a membranous structure that vibrates in response to sound, termed the tympanic membrane (eardrum). Medial to this membrane is an aerated cavity that houses three ossicles (bones) communicating tympanic membrane vibrations to a fluid-filled cavity, the cochlea. Attached to the ossicles are various ligaments and muscles that suspend the ossicles in the middle ear cavity and influence sound transmission. Cochlear soft tissue structures transform acoustically induced vibrations into trains of action potentials (Hudspeth, 1989; Hudspeth, 2005; Dallos, 2008; Ashmore et al., 2010). Action potentials then propagate as electrical signals along the nerve to the central auditory centers in the brain, where they are perceived as sound.

### 2.1 The middle ear cavity

The mammalian middle ear or tympanic cavity is an air-filled space in the petrous part of the temporal bone, housing the three ossicles, at least 13 ligaments, and two muscles. Subdivisions of the middle ear have been defined by spatial planes relative to the plane of the tympanic membrane: medially the mesotympanum, superiorly the epitympanum, inferiorly the hypotympanum, anteriorly the protympanum, and posteriorly the retrotympanum. The six “walls” of the tympanic cavity are the tegmental roof, formed by a thin bony plate covering the canal for the tensor tympani muscle and the tympanic antrum. They separate the middle cranial fossa from the tympanic cavity. The superior bulb of the interior jugular vein is inferior to the middle ear’s floor. The tympanic membrane and the epitympanic recess lateral bony wall form the lateral wall. The middle ear’s anterior wall separates the cavity from the carotid artery. The wall has an opening inferiorly to the Eustachian tube, a connection of the tympanic cavity to the pharynx. The epithelium near the Eustachian tube carries motile cilia, with a middle ear clearing function similar to the ciliary epithelium in the trachea. Disrupting the ciliary function may cause fluid buildup in the middle ear and subsequent hearing loss. The anterior wall also has a semicanal for the tensor tympani muscle. The posterior wall separates the tympanic cavity from the mastoid cells. The stapedius muscle enters through the posterior wall. The cochlea forms the medial wall.

### 2.2 The tympanic membrane

**FIGURE 2:**
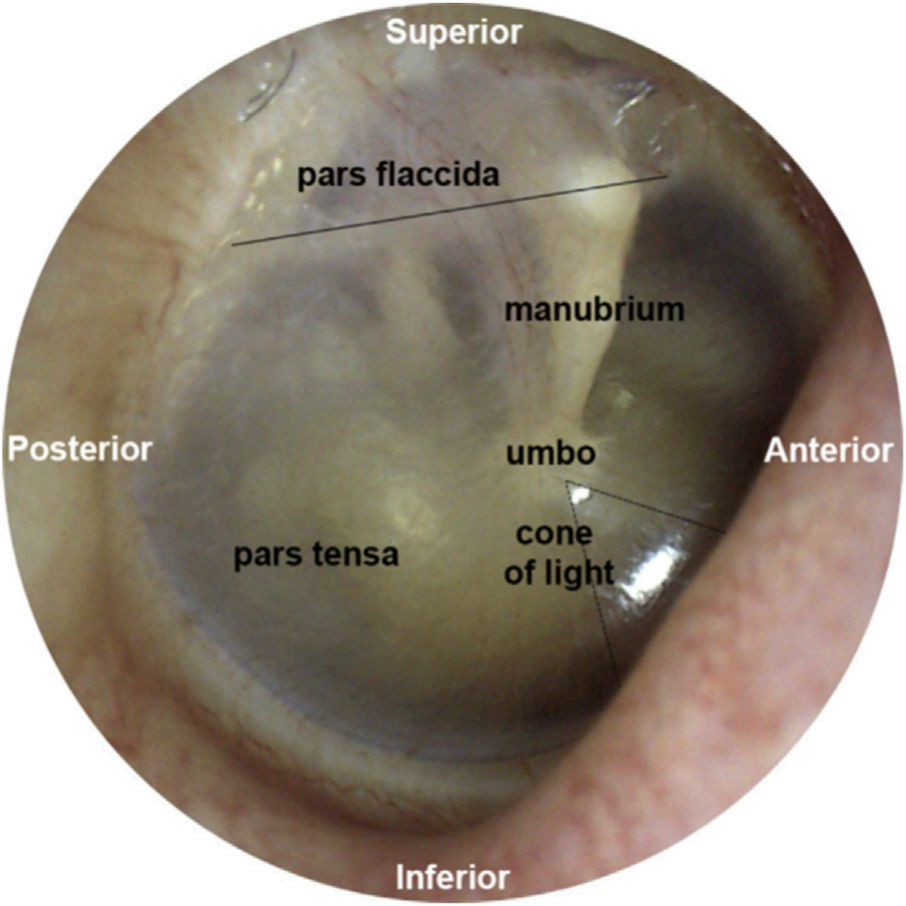
This figure was previously published in Brister et al. (2020a). The image of the middle ear is taken from the tympanic membrane of a right ear. Above the solid black line is pars flaccida, and below pars tensa. The umbo forms the tip of the manubrium. The reflection called the cone of light points toward the nose.

The tympanic membrane (Figure 2) terminates the external ear canal and forms the input to the middle ear. It can be directly visualized with a suitable light source and magnifier, for example, an otoscope. Already in 1832, Shrapnell described two parts of the tympanic membrane, pars tensa, and pars flaccida. Pars tensa is the stiff, inferiorly located section of the tympanic membrane (Figure 2) and is responsible for transmitting sound vibrations. The layers of this trilaminar membrane are the inner mucosal, the intermediate fibrous, and the outer epidermal layer. Tension by the middle ear ossicles and the ligamentous attachments to the ear canal wall at the tympanic membrane’s periphery hold pars tensa in place. Furthermore, smooth muscle arrays within the peripheral rim may have a role in maintaining the tension of the membrane (Henson et al., 2005). Superior to the manubrium (Figure 2), a flaccid section, pars flaccida, moves with pressure changes at frequencies well below the hearing range (Shrapnell, 1832a; Shrapnell, 1832b). Among mammals, pars flaccida varies in size. It is large in sheep, mice, and gerbils. In humans and cats, pars flaccida is relatively small and absent in guinea pigs (Decraemer and Funnell, 2008).

**FIGURE 3:**
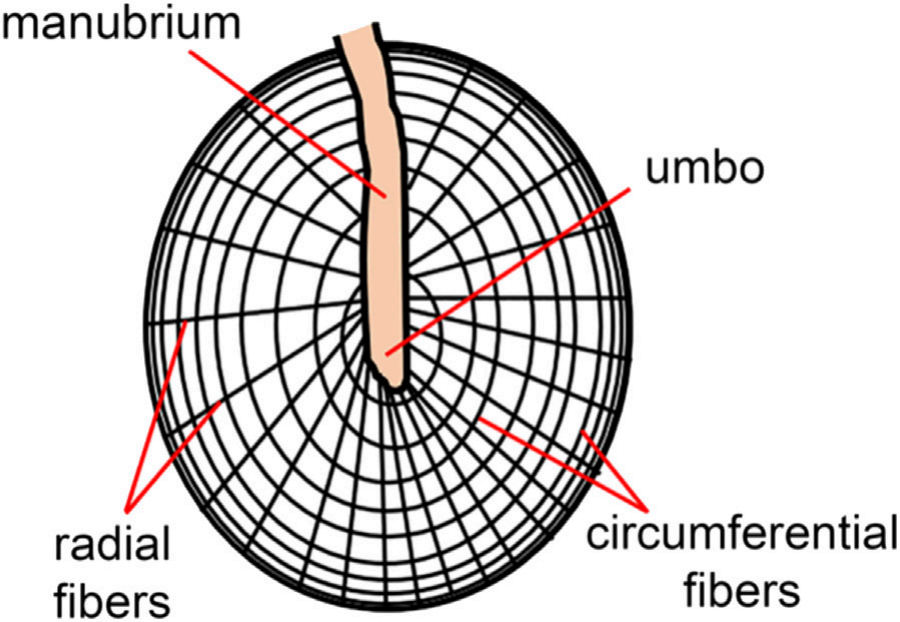
The skin of the ear canal forms the outer layer of the eardrum, and the mucous lining of the middle ear cleft forms the inner layer. The ultrastructure of the tympanic membrane shows the superficial radial and deeper circumferential fibers (Locke, 2013) made of collagen types I, II, and III (Stenfeldt et al., 2006).

The tympanic membrane is shaped like a conical horn and presumably acts as a waveguide, improving high-frequency sound transmission (Fay et al., 2006; Fields et al., 2018). Helmholtz suggested that middle ear structures pulling the tympanic membrane inwards explain its conical shape (Helmholtz, 1868). It is worth noting that this conical horn shape in mammals is more pronounced than in other vertebrate species (Fields et al., 2018). The shape of the tympanic membrane may also explain the larger bandwidth of mammalian hearing compared to other terrestrial vertebrates (Fields et al., 2018). As indicated by the sketch in Figure 3, radial and deeper circumferential fibers within the intermediate fibrous layer form the tympanic membrane’s conical feature (Locke, 2013). Radial fibers are predominant at the center of the eardrum, while circumferential fibers get denser towards the periphery (Fay et al., 2006). The fibers are made of collagen types I, II, and III (Stenfeldt et al., 2006) and provide structural integrity.

In 1949, von Békésy assessed the tympanic membrane’s stiffness using static bending tests with a calibrated probe (thin hair), reporting Young’s modulus to be 20 MPa (Von Békésy, 1949). Subsequent studies applied longitudinal tensile tests (Kirikae, 1960; Decraemer et al., 1980; Cheng et al., 2007; Luo et al., 2009) and nanoindentation testing on the tympanic membrane tissue sections to assess its stiffness (Huang et al., 2008; Daphalapurkar et al., 2009) or tested the whole membrane. Values of the tympanic membrane stiffness varied significantly (i.e., whole tympanic membranes, ranging from 2.1 to 300 MPa (Fay et al., 2005; Gaihede et al., 2007; Aernouts et al., 2012; Rohani et al., 2017), even when using the same testing method (i.e., longitudinal tensile tests: 0.4–58.9 MPa). This variability was due to different testing methods, specimen preparations, inter-specimen variation, and heterogeneity of the tympanic membrane and where the tested tissue samples were harvested (Rohani et al., 2017). However, measurements of the quasi-static regime (Cheng et al., 2007) and dynamic regime (Zhang and Gan, 2013) indicate that Young’s modulus of the tympanic membrane increases with increasing stress and increasing frequency (Lobato et al., 2022).

### 2.3 The ossicles and ligaments

**FIGURE 4:**
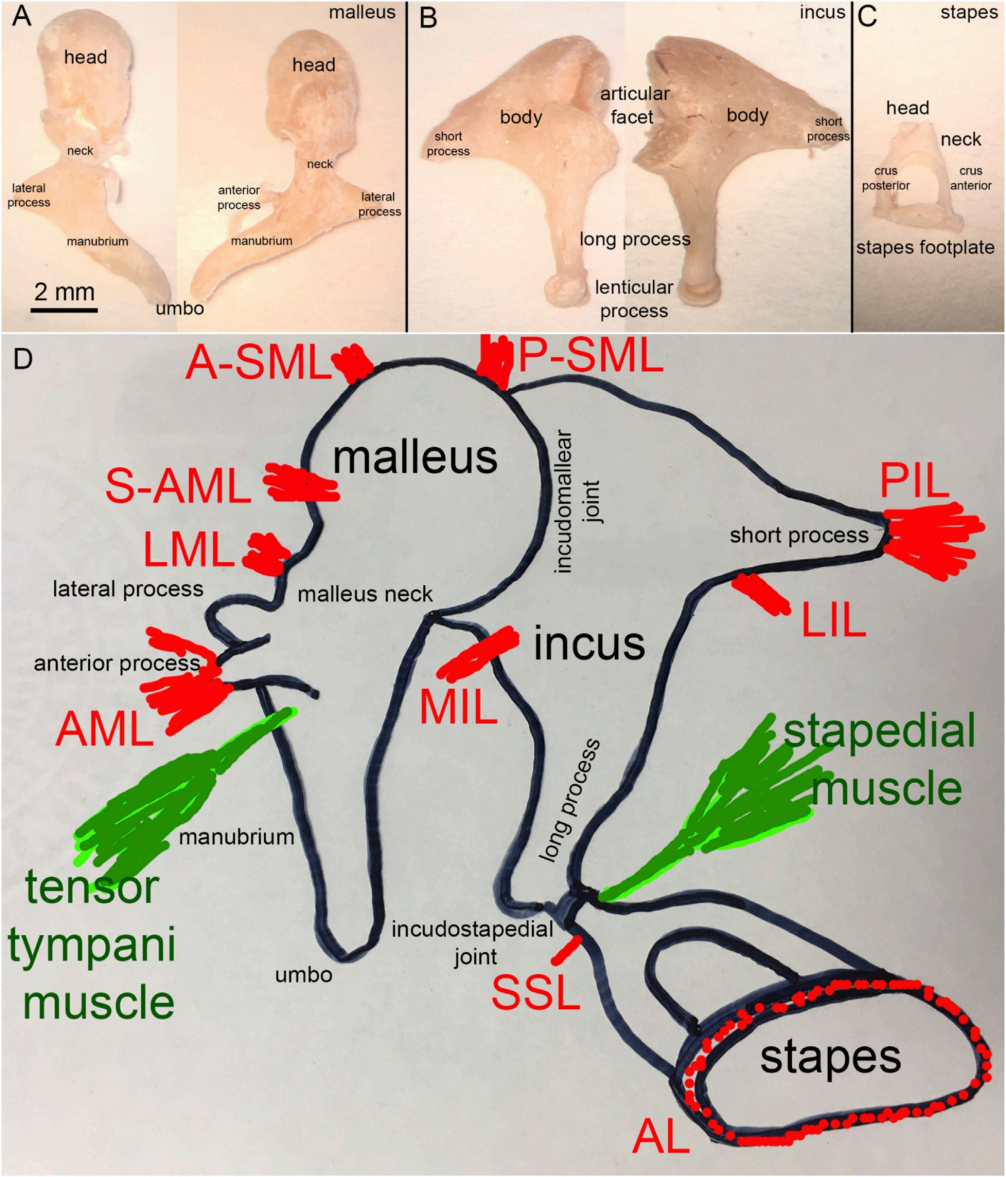
**(A)** Human malleus, **(B)** incus, and **(C)** stapes **(D)** Schematic representation of the attachment of the major middle ear ligaments.

Mammals have three middle ear ossicles: the malleus, incus, and stapes (Figure 4). The shape and size of the middle ear and its ossicles vary considerably among mammals and can be based on phylogeny, body size, and acoustic environment. Phylogenetically, mammals can be subdivided into monotremes, which lay eggs to replicate (e.g., Platypus), marsupials, which give birth to barely developed offspring (e.g., kangaroo), and placentals, where much of the development of offsprings occurs *in-utero* (e.g., humans). To functionally categorize the middle ears of mammals, Fleischer suggested six functional types: monotreme ears, therian ancestral ears, microtype ears, transitional type ears, freely mobile ears, and cetacean ear types (Fleischer, 1978; Mason, 2013). In the following, we focus on the freely mobile type found, for example, in humans, rabbits, guinea pigs, and chinchillas. Notably, a significant amount of recent data on the micromechanics of the middle ear originate from mice, which are considered microtype ears (Fleischer, 1978; Zhang et al., 2003; Mason, 2013). While mammals have three middle ear ossicles, the incus and malleus are fused in chinchillas and guinea pigs. Birds have a single middle ear ossicle. As discussed by several authors in the literature, the number of ossicles affects the animal’s hearing range, being largest in the animals with three middle ear ossicles (Fleischer, 1978; Puria and Steele, 2010; Mason, 2013; Fields et al., 2018).

#### 2.3.1 The malleus and its ligaments

Figure 4A shows a human malleus. The thick rounded head and thinner manubrium are the two major bony landmarks. The neck of the malleus is the contraction between the head and the manubrium. The back of the head forms the facet for the incudomalleolar joint. While Dahmann (1929) and Frank (1923) suggested a functional role for this joint, Bárány (1910) and Von Békésy (1939) assumed that no articular movement occurs at the incudomalleolar joint with the heads of the incus and malleus moving together as a rigid block. Just below the neck, at the beginning of the manubrium, two processes originate, the long process (anterior process or processus Folianus or processus gracilis) and the short process (lateral process or processus brevis) (Helmholtz, 1954). The long process points anteriorly and is covered by ligaments, while the short or lateral process attaches to the tympanic membrane (Figure 4D). The ligaments of the malleus are shown in Figure 4D. De Greef described 13 ligaments in human middle ears, of which seven attach to the malleus (De Greef et al., 2015). They are the anterior (AML), medial anterior (M-AML), superior anterior (S-AML), lateral (LML), posterior (PML), anterior superior (A-SML), and posterior superior (P-SML) mallear ligaments (De Greef et al., 2015). Ligaments surrounding the long process separate into the anterior mallear ligament (AML), which forms the anterior portion and attaches to a bony spine of the osseous tympanic ring (Helmholtz, 1954; De Greef et al., 2015). Thin portions of the ligament, which are not always present, are the S-AML or suspensory mallear ligament and the M-AML (De Greef et al., 2015). The posterior portion of the same ligament forms the PML and appears as an extension of the tympanic ring (Helmholtz, 1954; De Greef et al., 2015). PML and AML keep the malleus in position even after removing the other middle ear ossicles (Helmholtz, 1868). From axial compression tests, Young’s modulus of the human malleus and incus was 3.8 ± 0.5 GPa (Speirs et al., 1999). Micro-indentation testing of the rabbit caput malleus resulted in a stiffness of 16 ± 3 GPa, and of the rabbit collum malleus of 15.6 GPa, (Soons et al., 2010). Uniaxial tensile tests on the quasi-static regime revealed that the AML has Young’s modulus of ∼6 MPa at 0.5 MPa stress (Cheng and Gan, 2007; Cheng and Gan, 2008a; Cheng and Gan, 2008b). PML and AML also form a rotational axis for the malleus. The A-SML and P-SML prevent the malleus head from turning too far (Helmholtz, 1954; De Greef et al., 2015). The malleus attaches to the tympanic membrane with the manubrium, from the manubrium’s tip, the umbo (Figure 4A), to the lateral process.

#### 2.3.2 The incus and its ligaments

Figure 4B shows the incus. Anatomical landmarks of the incus are the short process, the long process, and the lenticular process. The latter attaches to the long process of the incus *via* a thin bony pedicle and articulates with the stapes at the incudostapedial joint. The head of the malleus articulates with the body of the incus. The incudomalleolar joint is a curved depression resembling a saddle (Helmholtz, 1868; Puria and Steele, 2010). It is biaxial, allowing movements in two planes.

Lauxmann et al. (2012) determined the load-deflection curves in humans in the lateral-medial and anterior-posterior directions. The results showed, on average, a rupture force of 894 mN in the anterior-posterior and 695 mN in the lateral-medial direction. Lauxmann et al. (2012) also measured micro-rupture forces with average values of 568 mN in the anterior-posterior and 406 mN in the lateral-medial direction. Short-term maximum forces with increased displacement were considerably larger (Lauxmann et al., 2012). Young’s modulus of the corpus of the rabbit incus was determined by micro-indentation and was 16.8 ± 3 GPa and 17.1 GPa for the crus of the incus (Soons et al., 2010). The ion composition of a gerbil incus showed spatial distribution patterns of chloride, calcium, potassium, and zinc specific for a gerbil at postnatal day 5 (Brister et al., 2020b). Biological key elements such as zinc indicate areas of active ossification (Brister et al., 2020b).

In humans, the average synovial-fluid-filled gap between the malleus and incus is 40–320 µm (Marquet, 1981; Sim and Puria, 2008; Puria and Steele, 2010). Four ligaments attach to the incus: the medial incudomalleolar ligament (MIML) and the posterior, medial, and lateral incudal ligaments (PIL, MIL, and LIL, respectively). The PIL is a short and strong ligament attaching the incus to the posterior wall of the middle ear cavity (Figure 4D). The PIL and AML also form the “axis of rotation” described by Helmholtz (1868), which we will present later in section 4.

#### 2.3.3 The stapes and its ligaments

Structures identified at the stapes are the head, the neck, the anterior and posterior crus, and the base (stapes footplate; Figure 4C). The stapes footplate inserts into the cochlear oval window and is sealed into it by the annular ligament (AL; Figure 4C), a ligament mechanically described as a viscoelastic material with nonlinear characteristics (Gan et al., 2011). Figure 4D shows the two ligaments attaching to the stapes, the superior stapedial ligament (SSL) and the annular ligament (AL).

**FIGURE 5:**
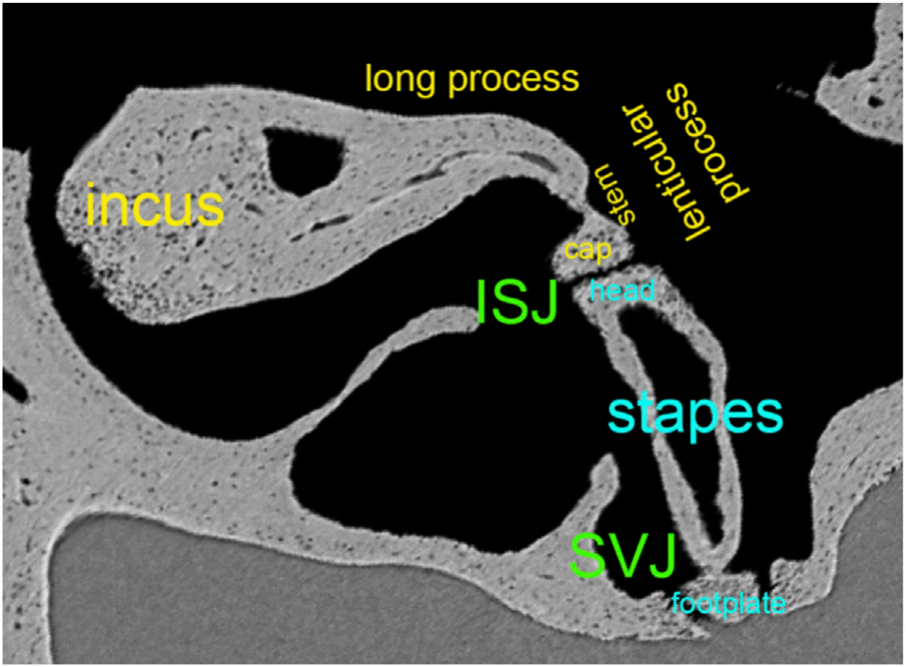
Synchrotron x-ray microtomography image from the left ear’s incudostapedial joint (ISJ) and stapediovestibular joint (SVJ) in a mouse. It shows the stem and cap of the lenticular process, the long process, and the head and neck of the stapes.

Mechanical properties of the AL vary among different sources and tests (Lynch et al., 1982; Waller and Amberg, 2002; Lauxmann et al., 2014; Zhang and Gan, 2014; Kwacz et al., 2015) with Young’s modulus of the ligament being 0.01 MPa under uniaxial tensile stress (Lynch et al., 1982), 0.031 MPa at 1 kHz under shear deformation in dynamic conditions (Zhang and Gan, 2014), and 1.1 MPa using atomic force microscopy (Kwacz et al., 2015). The AL bridges the gap between the stapes footplate and the bony margin of the oval window, the opening to the fluid-filled scala vestibuli of the cochlea, and forms the stapediovestibular joint (SVJ, Figure 5).

Figure 5 shows the convex lenticular process of the mouse incus and the concave stapes head, forming the smallest joint of the body, the incudostapedial joint (ISJ) (Karmody et al., 2009). It is a synovial joint with a joint capsule, cartilage, and synovial fluid (Ohashi et al., 2005; Wang et al., 2006). Reported mechanical properties of the human ISJ included Young’s modulus in uniaxial tension and compression, stress relaxation, and failure tests. Young’s modulus of the incus and stapes head were 1.41*10^10^ Pa (Zhang and Gan, 2011), 1.0*10^7^ Pa for the cartilage (Funnell et al., 2005). The synovial fluid had a bulk modulus of 2.2*10^9^ Pa and a viscosity of 0.4 Ns/m^2^ (Sasada et al., 1979; Fung, 1993). The failure force of the ISJ was, on average, 465 mN, the failure displacement 0.291 mm, the failure stress 3.19 MPa, and the failure stretch ratio 2.04 (Zhang and Gan, 2011). After modeling the data using the Ogden model to describe the nonlinear behavior of the joint capsule, Zhang and Gan concluded that the ISJ is a viscoelastic structure with a nonlinear stress-strain relationship.

Compression tests of the entire ISJ resulted in a complex modulus of about 1.17 MPa in the quasi-static regime (Zhang and Gan, 2013) and about 1.14 MPa at 1 Hz in the dynamic regime (Jiang and Gan, 2018). Dynamic studies suggest that the complex modulus of the ISJ and the stiffness of the AL increase with stimulation frequency (Zhang and Gan, 2014; Jiang and Gan, 2018).

Most middle ear ligaments have been described in the previous sections, but more may exist. Middle ear ligaments are not consistently present or identifiable in all species because they are often embedded by larger mucosal folds and stands. Their number also varies among mammals. Of the thirteen, the six primary middle ear ligaments in humans holding the chain of middle ear ossicles in place are the ALM, LML, A-SML, P-SML, the PIL, and the AL (Ludwig, 1852; Gray, 1878; Decraemer and Khanna, 1994; Gan et al., 2007a; Dai et al., 2007). Across most mammalian ears, the PIL and the AML, or a thin, bony analog for the AML, are the two major attachments consistently present (Kobayashi, 1955b; a; Mason, 2013). They have been suggested to limit the direction of motion and define the rotational axes of the ossicular chain (Fleischer, 1978; Lavender et al., 2011; Mason, 2013). However, published experimental data has not historically supported this putative role. The severing of one of these ligaments did not substantially change the middle ear transfer function (Hato et al., 2001; Gan et al., 2007a; Dai et al., 2007). However, a recent study showed that severing more than one of the suspensory ligaments produced a static shift in the position of the ossicles and reduced sound transmission to the cochlea (Brister et al., 2021).

### 2.4 The middle ear muscles and tendons

Two muscles, the tensor tympani and the stapedius muscle (Figure 4D) attach to the ossicles and reduce sound transmission through the middle ear when contracted (Wever and Vernon, 1955; Galambos and Robert, 1959; Simmons et al., 1959; Hilding and Fletcher, 1960; Terkildsen, 1960; Klockhoff, 1961; Djupesland and Zwislocki, 1973). The tensor tympani muscle originates from the cartilaginous portion of the auditory tube and the sphenoid and inserts at the neck of the malleus. Neurons near the trigeminal motor nucleus innervate the muscle (Strutz et al., 1988). Studies on the cat’s middle ear show that the tensor tympani muscle can pull about 3.5 g (∼34 mN, Wever et al., 1955). Contraction of the tensor tympani muscle pulls the TM and malleus into the middle ear air space, increasing the stiffness of the TM (Nuttall, 1974) and the static pressure within the middle ear.

The stapedius muscle, whose exact role is not fully understood, originates from the pyramidal eminence, a conical projection in the middle ear behind the oval window, and inserts at the neck of the stapes. Some studies show that it contracts in response to vocalization and swallowing, reducing the transmission of internal noises to the cochlea. It also contracts in response to loud noises and likely reduces sound transmission to the cochlea by up to 15 dB. The reduction in response amplitude depends on the stimulation frequency (Borg, 1968; Borg and Møller, 1968). Experiments in cats showed that the stapedius muscle pulls about 1.6 g (∼15.7 mN, Wever et al., 1955). The onset time of this response in the contralateral ear is about 15 ms (Galambos and Robert, 1959; Simmons et al., 1959), which means that the reflex does not protect from high-level impulse noises such as gunshots. The duration of the acoustic reflex is about 300 ms, which means that the reflex provides limited protection for extended noise exposures (Djupesland, 1964). Nerve fibers running along the facial nerve, originating outside the traditionally facial nucleus, innervate the stapedius muscle (Strutz et al., 1988).

Uniaxial tensile tests on the quasi-static regime revealed that the stapedial tendon and tensor tympani tendon showed a stiffness of 6 MPa at 0.5 MPa stress, similar to that of the AML (Cheng and Gan, 2007; Cheng and Gan 2008b; Cheng and Gan 2008a).

## 3 Effects of development on middle ear structure

Studies of the development of the middle ear examine how the structures form over time until they reach maturity (Dreyfuss, 1893; Broman, 1899; Jenkinson, 1911; Goodrich, 1915). Early experimental results on the middle ear ossicle development suggested that the stapes originates from the hyoid arch’s upper end (Dreyfuss, 1893; Broman, 1899; Jenkinson, 1911; Goodrich, 1915). Histological studies of the early 20th century continued to clarify the developmental stages of the ossicles without reaching a consensus (Anson, 1942; Cauldwell and Anson, 1942; Anson et al., 1948; Richany et al., 1954a; Richany et al., 1954b). More recent work suggests a dual origin for the stapes (Thompson et al., 2012; Anthwal and Thompson, 2016), with the head, crura, and inner footplate originating from the neural crest of the second arch; the outer footplate having a mesodermal origin (O’gorman, 2005; Thompson et al., 2012). The malleus and incus originate from the Meckel’s cartilage and separate later during development (Anthwal and Thompson, 2016; Powles-Glover and Maconochie, 2018).

Unlike the rest of the bones in the body, in humans, the middle ear ossicles are full size at birth (Powles-Glover and Maconochie, 2018). Once completely ossified, their high stiffness makes the ossicles more brittle than the skeletal bones of the skeleton (Currey, 1999a; Currey, 1999b; Kuroda et al., 2021). However, their location within the skull protects them from impact trauma. Their high stiffness benefits sound conduction (Currey, 1999a; Currey, 1999b), as minimal energy is lost in elastic deformations. Auditory osteoblasts, a novel osteoblasts subtype, produce collagen type I and type II as scaffolding for their bone matrix (Kuroda et al., 2021)- a phenomenon only found in the middle ear ossicles - have been recently considered the responsible cells for the ossicles high mineralization (Kuroda et al., 2021). The ossicles’ bone tissue presents both empty and abnormal levels of hyper-mineralized lacunae already at early ages (Rolvien et al., 2018). The early degeneration of the osteocytes without subsequent bone remodeling is a remarkable phenomenon of the ossicles (Marotti et al., 1998; Palumbo et al., 2012; Rolvien et al., 2018) and might be responsible for the hyper-mineralization of the empty lacunae. The lack of bone remodeling in the ossicles is thought to be a mechanism of the ear bones to preserve their structure, which would otherwise go through shape adaptation as a response to the mechanical stimuli (Duboeuf et al., 2015; Rolvien et al., 2018). In the spiral ligament and inner ear space, elevated osteoprotegerin (OPG) levels (>1000 x long bone levels) (Zehnder et al., 2005; Bloch and Sørensen, 2010) have been found and suggested to diffuse into the otic capsule and propagate to the ossicular chain (Bloch and Sørensen, 2010). OPG, a powerful inhibitor of bone turnover (Theoleyre et al., 2004), has been termed audioprotegerin for its suggested role in maintaining the integrity of the ossicular chain and otic capsule by suppressing osteoclast survival and activation (Kanzaki et al., 2006).

## 4 Middle ear function and sound transmission

Sound propagates in the air *via* the continuous exchange of kinetic and potential energy. At constant temperature and air pressure, the sound wave will propagate without reflection due to the homogeneous nature of the medium. The sound velocity is sufficient that the transfer of sound is considered adiabatic. For a sound initiated with a force denoted by a sound pressure, P, the flow of the sound wave (volume velocity), U, produced by this sound pressure depends on the acoustic impedance, Z, of the medium, i.e.,

$$U=\frac{P}{Z}$$ (1)

this being the familiar Ohm’s Law expressed in acoustico-mechanical terms. The characteristic impedance of air, *Z*_0_, is given by

$$Z_{0}=\varrho *c$$ (2)

where ρ is the density of air and c is the velocity of sound.

A change in the properties of the medium, through which sound transfers, constitutes an acoustic impedance change, resulting in sound being reflected at the boundary of this impedance change. The amount of reflection calculates according to Fresnel (1823) as follows:

$$R=\frac{\left(Z_{2}-Z_{1}\right)}{\left(Z_{2}+Z_{1}\right)}\;,$$ (3)

where R is the pressure reflectance, Z_1_ is the acoustic impedance of the first medium, and Z_2_ is the acoustic impedance of the second medium, with $Z_{1}\leq Z_{2}$ . R ranges in value from 0 to 1, with 0 being no reflection and 1 being total reflection.

Thus, a system with significantly different Z_1_ and Z_2_ (i.e., the impedance mismatch between air and fluid) would exhibit more than 99.9% energy losses due to reflections at their interface (Wever et al., 1954; Wever and Lawrence, 1954; Killion and Dallos, 1979). Killion and Dallos (1979) computed the impedance mismatch to be larger than 50 dB at 100 Hz. The semi-rigid ossicular chain connects the large tympanic and small oval window membranes. The surface area ratio between these two membranes is the basis for a purported hydromechanical transformer that decreases the sound-induced displacements at the oval window membrane while increasing the sound pressure (Wever et al., 1954; Killion and Dallos, 1979; Rosowski, 2010). Killion and Dallos (1979) concluded that the middle ear alone could not compensate for the losses; rather, it is the combined action of the outer ear resonance and the middle ear. In the human hearing system, the pressure reflectance is close to 1 at low frequencies but becomes much lower at frequencies around 1–4 kHz and increases once again at higher frequencies (∼1 above 10 kHz). The middle ear most efficiently transmits acoustic energy at these intermediate frequencies, where human hearing is most sensitive.

## 5 Kinematics of the middle ear

### 5.1 The incudomalleolar joint

**FIGURE 6:**
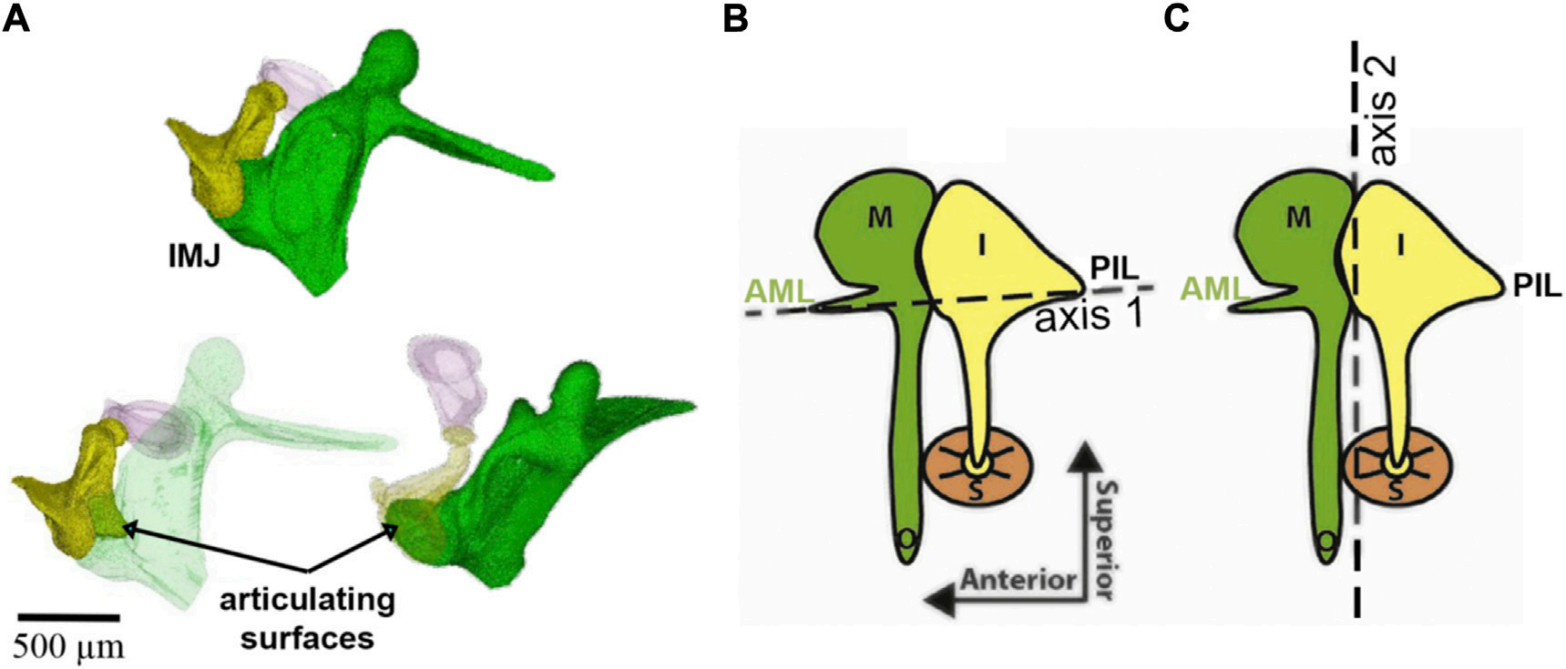
Panel **(A)** shows the incudomalleolar joint and articulating surfaces of a mouse ear. A synovial-fluid-filled gap between malleus and incus exists (Ihrle et al. (2017). Panels **(B)** and **(C)** were taken from (Rosowski et al., 2020) and modified. The malleus (M), incus (I), stapes (S), anterior mallear ligament (AML), and posterior incudal ligament (PIL) are shown. The dotted lines show two axes of ossicular rotation, one in panel **(B)** and one in panel **(C)**. The axis in **(B)** is axis 1 at low frequencies in the freely-mobile ear (Helmholtz, 1868; Fleischer, 1978; Puria and Steele, 2010); panel **(C)** shows the freely-mobile ear with the high-frequency axis 2 of rotation.

Helmholtz, while manipulating the manubrium with a needle tip, suggested that the middle ear is a system of levers that rotates around a single axis formed on one side by the AML and the dorsal fibers of the LML and on the other side by the PIL (Figure 6). It was von Békésy who demonstrated the ability of the incudomalleolar joint to move the incus biaxially (Von Békésy, 1960). Specifically, at physiological sound levels at the tympanic membrane, IMJ motion changes with frequency. While at frequencies at the low end of the hearing range, rotations occur around axis 1, at frequencies at the higher end of the hearing range, rotations are around axis 2 (Figure 6).

The faces of the incudomalleolar joint are medial and superior to the joint’s rotational axes (Figure 6), and one can infer that the incus slides up and down relative to the malleus. Small inside-outside (direction of the manubrium tip movement) and front-to-back (direction of the stapedius muscle) movements may also occur. Upward movement of the incudomalleolar joint tilts the incudal body around the posterior incudal ligament, rotating the lenticular process anteriorly and superiorly, leading to a reversal of the movement at the incudomalleolar joint. This also uncouples the incudostapedial joint from large movements caused by pressure changes originating from events such as atmospheric elevation changes, sneezing, and swallowing, maintaining the input of the sound energy to the ear under varying environmental conditions and perhaps protecting the inner ear.

### 5.2 The incudostapedial joint

Karmody et al. (2009) showed that the motion of the incudostapedial joint depends on the sound intensity level. The incus and stapes move synchronously in a lateral to medial motion at low-intensity sounds. In contrast, the motion of the joint at high-intensity sounds is mainly dictated by the stapedial muscle pulling the stapes posteriorly and perhaps protecting the inner ear from hazardous sound (Karmody et al., 2009).

### 5.3 The stapediovestibular joint

Sound energy is transmitted to the fluid-filled cochlea at the stapediovestibular joint by the piston-like stapes movements at the low-frequency end of the hearing range (Von Békésy, 1960; Gyo et al., 1987; Suzaki et al., 1997). For frequencies at the upper end of the hearing range, the stapes movements adopt lateral components resulting in complex rocking, hinge-like motions (Suzaki et al., 1997).

In addition to the described malleus movement induced by large mechanical displacements with high-pressure changes or by needle manipulation of the manubrium, other modes of vibrations exist as have been demonstrated with more sensitive methods, lately with optical coherence tomography (OCT). The ossicular chain moves along a circular path with a center of rotation above the ossicles resulting in a piston-like umbo and stapes movement. On the other hand, such a pattern would require the middle ear ossicle joints to be fixed, and no gain would be obtained from the lever action. Consequently, the middle ear could be replaced by a single ossicle, as is done in many middle ear replacement surgeries.

## 6 Middle ear mechanics explained by experiments

The motion of the tympanic membrane in response to sound has been studied using various measurement techniques, including capacitive probes (Von Békesy, 1941; Von Békésy, 1960), laser doppler velocimetry (LDV) (Schops et al., 1987; Stasche et al., 1994; Goode et al., 1996; Aarnisalo et al., 2009; Szymanski et al., 2009; Aarnisalo et al., 2010; Kunimoto et al., 2014; Wang et al., 2016; Jiang et al., 2019), speckle contrast interferometry (Lokberg et al., 1980; Wada et al., 2002), holography (Tonndorf and Khanna, 1970; Tonndorf et al., 1971; Tonndorf and Khanna, 1971; Furlong et al., 2009; Rosowski et al., 2009; Cheng et al., 2010; Cheng et al., 2013; Khaleghi et al., 2013; Dobrev et al., 2014; Khaleghi et al., 2015a; Khaleghi et al., 2015b; Tang et al., 2019; Tang et al., 2021), and optical coherence tomography (OCT) (Subhash et al., 2012; Chang et al., 2013; Burkhardt et al., 2014; Dobrev et al., 2016; Park et al., 2016; Razavi et al., 2016; Jeon et al., 2019a; Jeon et al., 2019b). More details about the methods are reported in the appendix.

### 6.1 Biomechanics of the tympanic membrane

**FIGURE 7:**
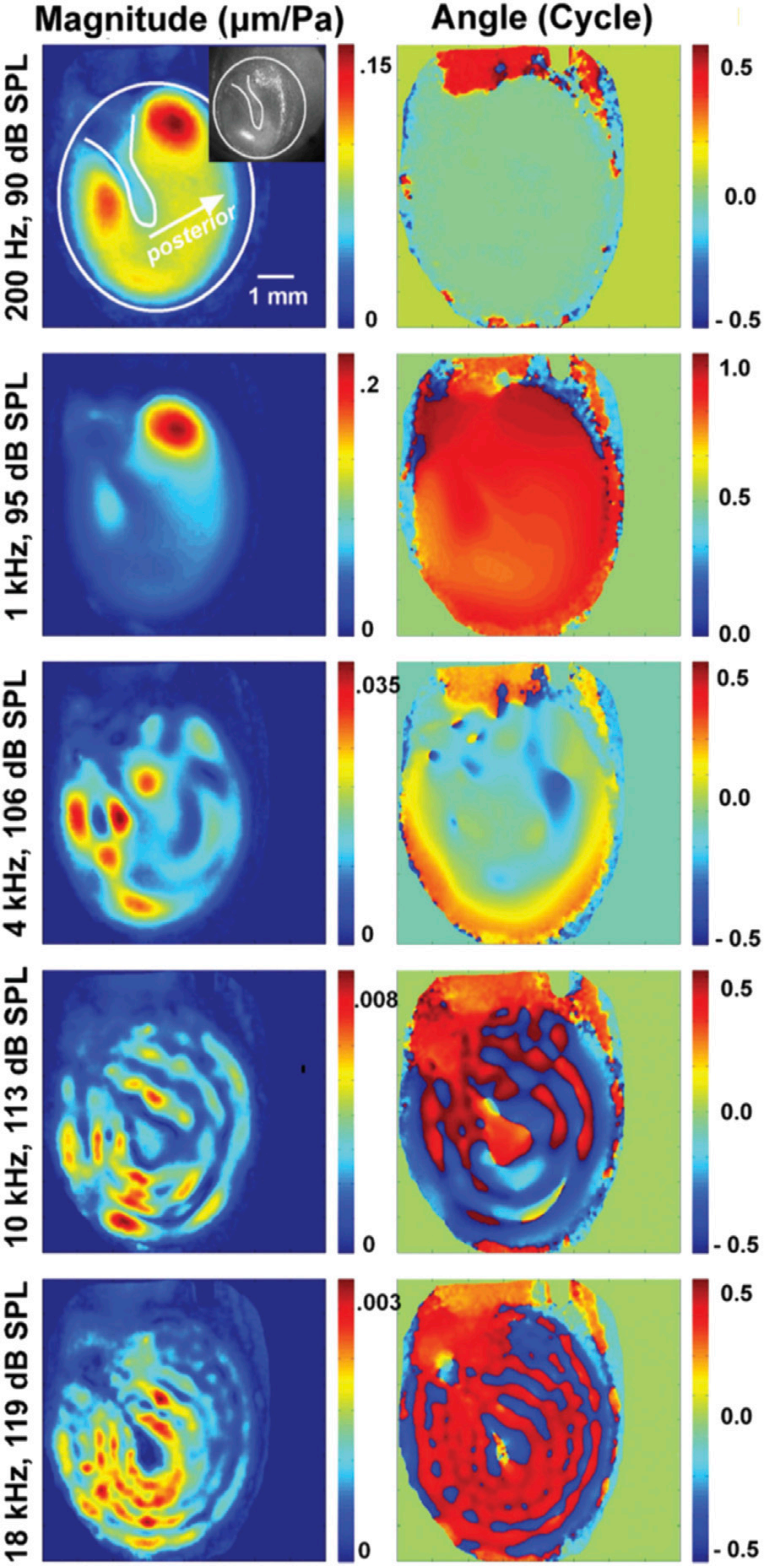
This plot is from Cheng et al. (2013), showing the change in vibration patterns and phase of vibrations of the tympanic membrane obtained with holographic measurements.

Early holographic measurements suggested the tympanic membrane vibrations to be simple at low frequencies and become complex, with quasi-independent vibration patterns occurring across the membrane at higher frequencies (Tonndorf and Khanna, 1970). Holography also revealed that the tympanic membrane moves less at the attachment to the manubrium of the malleus than at other locations (Figure 7). However, it is not clear how these localized vibrations relate to sound transfer to the middle ear.

**FIGURE 8:**
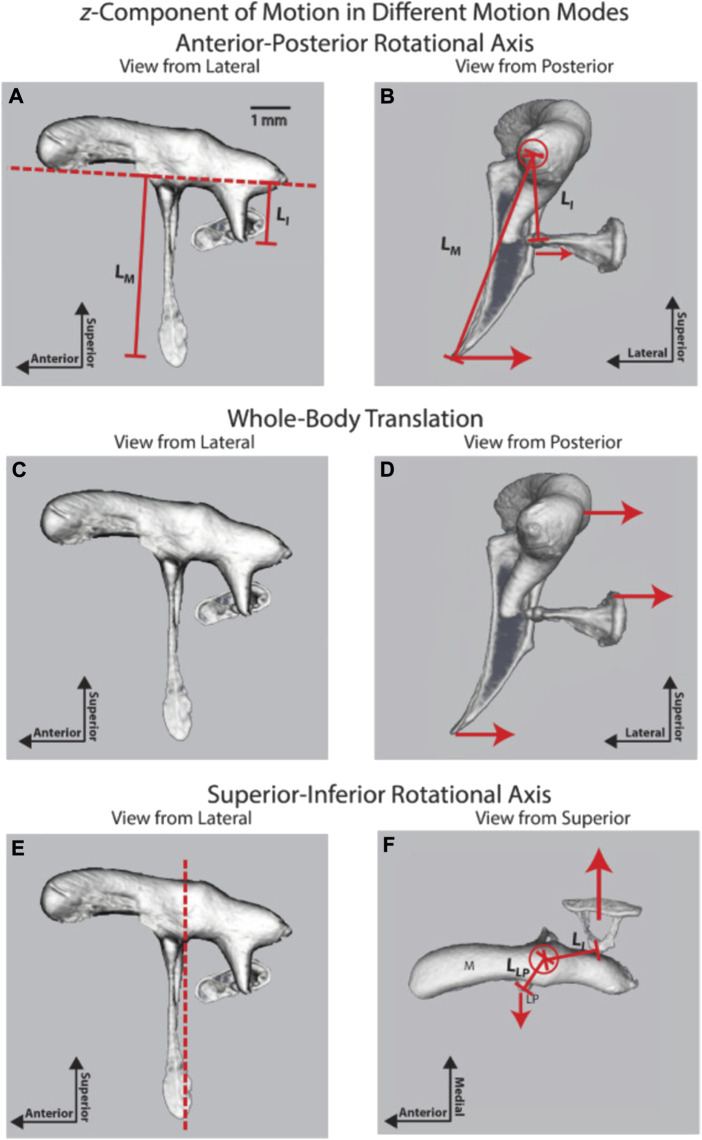
Three ossicular motion modes are described by Rosowski et al. (2020); reprinted with permission. **(A)** lateral and **(B)** posterior view of the anterior-posterior rotational axis **(C)** lateral and **(D)** posterior view of the whole-body translation, and **(E)** lateral and **(F)** posterior view of the superior-inferior rotational axis.

At the time of writing, there is some dispute regarding the location on the eardrum where the motion is most representative of sound transmission. A parsimonious explanation would be that the focal point is at the location of the umbo, the location that would represent the throat of the conical horn, and the point of maximum displacement of the eardrum. Note that the ligament that surrounds the eardrum and attaches to the ear canal wall has elasticity, so the eardrum is not rigidly attached to the canal wall. Contrary to this explanation are eardrum motion measurements, showing multiple local vibrations across the membrane (Figure 8) that do not propagate across the surface (Cheng et al., 2010; Cheng et al., 2013).

### 6.2 Biomechanics of the middle ear ossicles

As described above, an early understanding of middle ear mechanics is based on the assumption that a rotational axis exists parallel to a line through the AML, the incus, malleus, and PIL (Figure 6B). The ossicles rotate around this axis and result in a piston-like movement of the stapes footplate, transmitting the acoustical energy to the fluid-filled scala vestibuli. The length of the manubrium and the long process of the incus would introduce lever action in addition to the surface differences of the tympanic membrane and the stapes footplate. The existence of this rotational axis was confirmed in multiple measurements of middle ear vibration in humans, cats, gerbils, and chinchilla at stimulation frequencies at the lower end of the hearing range (Helmholtz, 1868; Dahmann, 1929; Von Békesy, 1941; Von Békésy, 1960; Guinan and Peake, 1967; Hüttenbrink, 1992; Rosowski et al., 1999; Decraemer and Khanna, 2004; Decraemer et al., 2014; Rosowski et al., 2020). As shown in Figures 8A,B, in chinchilla, the middle ear ossicles move in phase on one side of the rotation axis for stimulus frequencies below 2 kHz, and the displacement of the ossicles increases with the distance from the rotational axis (Rosowski et al., 2020). The movement patterns are complex for higher frequencies, with an additional axis of vibration emerging (Figures 8E,F). Fleischer (1978) was a strong proponent of additional axes of rotation and proposed two major axes, perpendicular to each other, for the incudomalleolar joint. In his view, the rotation around axis 1 (Figure 6B) determines the ossicle rotation at low frequencies, where the stiffness of the ossicular support limits the motion. However, in species such as mice and rats, he proposes a second axis of rotation (axis 2, Figure 6C) perpendicular to axis 1, running through the incudomalleolar joint producing an out-of-phase movement of the manubrium of the malleus and the incus. The results obtained from mouse middle ears with capacitive probes (Saunders and Summers, 1982) and laser doppler velocimeter (Dong et al., 2013) and from chinchilla middle ears using OCT (Rosowski et al., 2020) supported this view. On top of the rotation around axis 1, a whole-body translation of the ossicles was observed in the chinchilla between 3 and 8 kHz (Figures 8C,D) (Rosowski et al., 2020). A similar whole-body translation has been described for cats and humans at frequencies above 1–2 kHz (Decraemer and Khanna, 1994).

An additional mode of vibration relates to the bending of the manubrium (Rosowski et al., 2020), which was identified by the phase differences and vibration amplitudes along the manubrium. Bending of the manubrium has been explained by the distributed mass and stiffness along the manubrium and has also been identified in cats (Funnell et al., 1992; Decraemer and Khanna, 1995).

As described above, measurements of the middle ear revealed no simple malleus or incus movements around a simple axis but rather complex vibrations, including translations of the entire ossicular chain, which change with the frequency of the stimulus. It remains unclear how the stapes couples the complex vibrations to the fluid-filled cochlea. Studying stapes micromechanics is challenging because of the limited access to the ossicle: only a small portion of the crura, the neck, and the head can be imaged for measurements. Typically cadaveric preparations were used for the measurements. Such measurements revealed that stapes vibrations were driven by a piston-like motion at the low end of the hearing range, while rotary motions along the short and long axis of the footplate predominate at higher frequencies. Those patterns were found in all the animal species examined, such as humans (Heiland et al., 1999; Hato et al., 2003), cats (Guinan and Peake, 1967), and gerbils (Decraemer et al., 2007).

## 7 Middle ear function explained by models

### 7.1 Lumped-parameter models of the middle ear

Mathematical models have been used to further understand how the middle ear works. The simplest of these models are lumped-parameter models where the function of the middle ear is modeled by an electrical equivalent circuit, with the electrical circuit parameters defined in terms of their acoustic impedance properties. For this model type, inductors represent acoustic mass, capacitors represent acoustic compliance, and resistors represent acoustic resistance.

**FIGURE 9:**
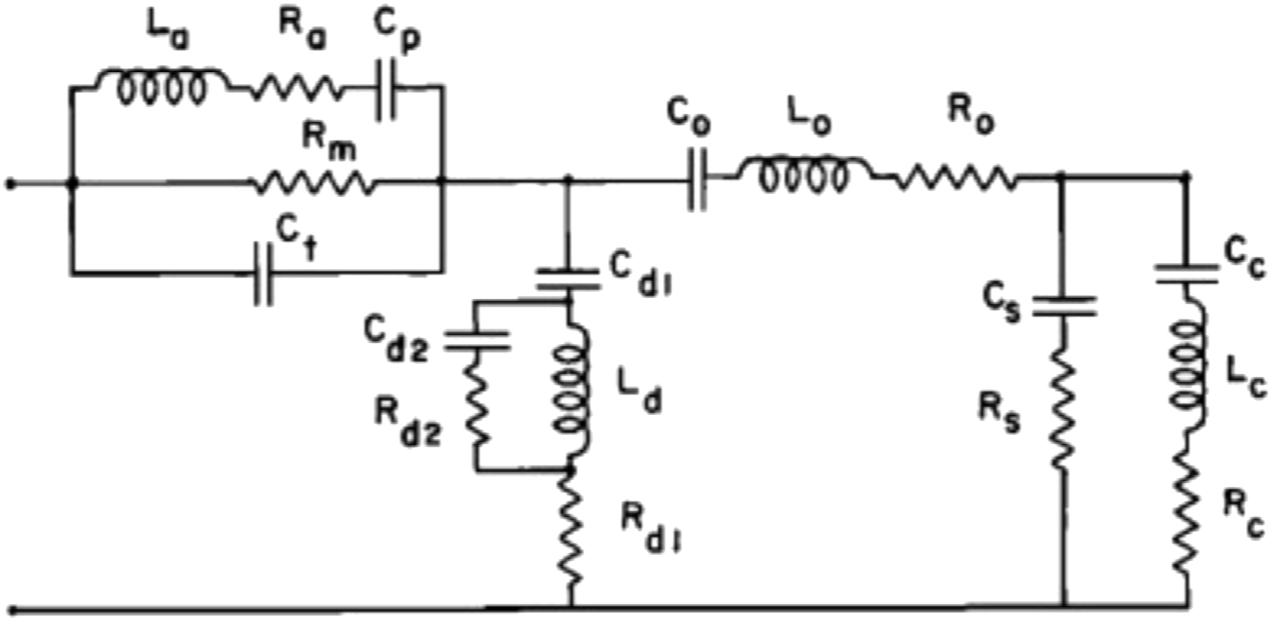
Electric analog of the middle ear; reprinted with permission from Zwislocki (1962). Electric elements are L for inductance, R for resistance, and C for capacitance. Elements with subscripts *a, p, m*, and *t* represent the middle ear cavities, those with *d* represent a portion of the tympanic membrane, and those with *o* represent the mallear complex.

One of the earliest and most cited models of this type was reported by Zwislocki (1962). This model represents the acoustic input impedance of the middle ear and includes elements for the middle ear cavities (antrum and tympanum), and for the tympanic membrane, ossicles, and cochlea, respectively, and the two shunts representing sound reflected back to the ear canal without reaching the cochlea due to non-ossicular coupling or an impedance discontinuity (Figure 9). This model reasonably describes the acoustic input impedance of the normal human ear up to about 2 kHz (Zwislocki, 1962).

Kringlebotn (1988) extended Zwislocki’s model by incorporating the ear canal as a rigid-walled tube, a lossy transmission line terminated by a load impedance (the middle ear model), and by adding circuit elements to represent the attachment of the eardrum to the walls of the ear canal, primarily an elastic attachment. Withnell and Gowdy (2013) found Kringlebotn’s model to reasonably describe experimental data from six human ears up to 6 kHz (the upper frequency limit of their study). Lumped parameter models have a limited frequency range of operation and cannot describe the frequency range of human hearing. Extending the frequency range requires incorporating transmission lines (Puria and Allen, 1998; O’connor and Puria, 2008; Fields et al., 2018).

### 7.2 Distributed parameter models

Distributed parameter systems have long described middle ear function, with the earliest studies dating back to the late 20th century (Funnell and Laszlo, 1978; Rabbitt and Holmes, 1986; Lesser et al., 1988; Wada et al., 1992). Unlike lumped parameter models described by ordinary differential equations, distributed parameter models predict the behavior of state variables in several independent coordinates using sets of partial differential equations (PDEs), boundary conditions, and initial conditions, characterizing the motion of complex, more realistic geometries and anatomical structures (Wouwer, 2009), thus overcoming the limitations of the lumped parameter models (Parent and Allen, 2007).

Most models at the time were in the frequency domain (Funnell and Laszlo, 1978; Rabbitt and Holmes, 1986). However, Parent and Allen implemented a remarkable distributed parameter model of the cat tympanic membrane in the time domain (Parent and Allen, 2007). This model simulated the frequency-independent delays of the ear canal and tympanic membrane observed in previous studies (Puria and Allen, 1998) as well as the propagating wave on the tympanic membrane surface. Shortly after, Goll and Dalhoff (2011) modeled the tympanic membrane of the guinea pig as a string with distributed force. They suggested that the effective area of the tympanic membrane depends on the frequency and is different for forward and reverse transduction (Goll and Dalhoff, 2011).

In their first finite element (FE)-based middle ear models, Funnell and Laszlo (1978) investigated the effects of the curved conical shape of the tympanic membrane of cats. In the following years, others assessed the effects of their elastic modulus changes on vibration shapes (Lesser et al., 1988), vibration patterns at different frequencies (Williams and Lesser, 1990; Wada et al., 1994), and predicted membrane rupture (Stuhmiller, 1989). The assumptions made experimentally by Tonndorf and Khanna in the 70s (Tonndorf and Khanna, 1970) were supported by these models in the 90s, confirming that the vibration patterns of the eardrum at low frequencies are simple but become complex with increasing frequency (Williams and Lesser, 1990). Wada et al. (1992) included the ossicles and cochlear impedance when modeling the tympanic membrane. Similarly, Ladak and Funnell (Ladak and Funnell, 1996) added explicit representations of the ossicles and cochlear load to the model implemented by Funnell and Laszlo (Funnell and Laszlo, 1978). Blayney et al. (1997) further modified the model to simulate the mechanics of sound transmission following stapedotomy by introducing a prosthesis to the model and adding pathological conditions. A significant improvement of these models came when Koike et al. (2002) further added anatomical structures such as tendons, ligaments, the incudostapedial joint, cochlear load, the external auditory canal, and the middle ear cavity, which allowed simulating complex ossicular vibrations (Koike et al., 2002). As a result, they observed that the middle ear cavities suppressed the tympanic membrane’s vibration amplitude at low frequencies (below 1.5 kHz) but did not alter the tympanic membrane’s vibration modes.

Gan et al. (2002) similarly added these structures to their FE model. They reported that the maximum displacements of the eardrum and stapes footplate happened at 3 and 4 kHz when applying the sound pressure at the entrance of the auditory meatus.

**FIGURE 10:**
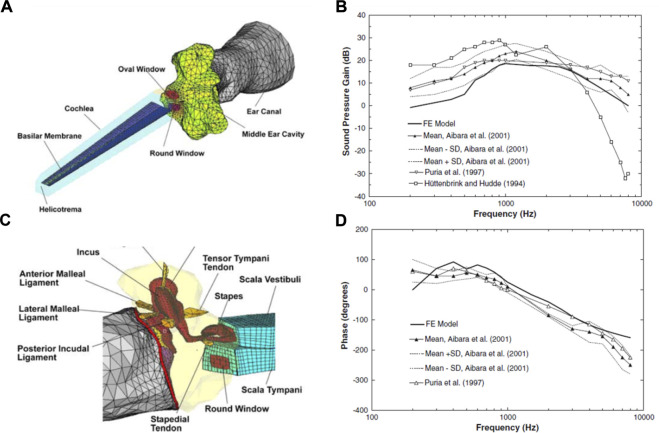
**(A)** Finite element model (FEM) of the middle and inner ear. Developed by Gan et al. (2007b). **(B)** Connection of the middle ear with the inner ear. Model derived pressure gain of Gan et al. (2007b) compared with others in the literature, showing **(C)** magnitude and **(D)** phase angle. Modified from Gan et al. (2007b).

Until 15 years ago, the middle ear and inner ear mechanics were modeled separately, with those modeling the middle ear adding solely the cochlear load to the stapes (Koike et al., 2002; Gan et al., 2004; Gan et al., 2006), and those modeling cochlear mechanics isolating the inner ear from the middle ear (Kolston and Ashmore, 1996; Böhnke and Arnold, 1999). Gan et al. (2007b) were the first to integrate both systems by adding an uncoiled cochlear model consisting of two straight fluid channels separated by the basilar membrane to their previously developed middle ear model (Gan et al., 2006). This was the first FEM in the field to model the entire human ear to study acoustic-mechanical transmission (Figure 10). Their model predicts the displacements of the tympanic membrane, stapes footplate, and round window, which generally agrees with experimental data (Gan et al., 2007b). They predicted and compared with previous models the sound pressure gain of the middle ear, measuring the ratio of the scala vestibuli to the pressure of the auditory meatus on the surface of the tympanic membrane (Figure 10).

Despite the great achievement of integrating cochlear structures into the models, subsequent models developed to study middle ear mechanics mostly continued to model the middle ear structures only, often accompanied by a cochlear load, as it considerably reduces computational costs. Lee et al. (2006) increased the models’ geometrical accuracy by introducing high-resolution computed tomography (CT) to derive FE models of the middle ear. This new approach brought new insights to the field, where up to that time, model geometry was based on destructive histological sections, anatomical data reported in the literature, or data directly measured from temporal bones (Zhao et al., 2009), allowing, therefore, to simulate middle ear pathologies with abnormal geometrical parameters, including tympanic membrane changes in stiffness or with perforations, middle ear cavity alterations (e.g., middle ear effusion) and disorders of the ossicular chain (e.g., stapes fixation) (Zhao et al., 2009).

**FIGURE 11:**
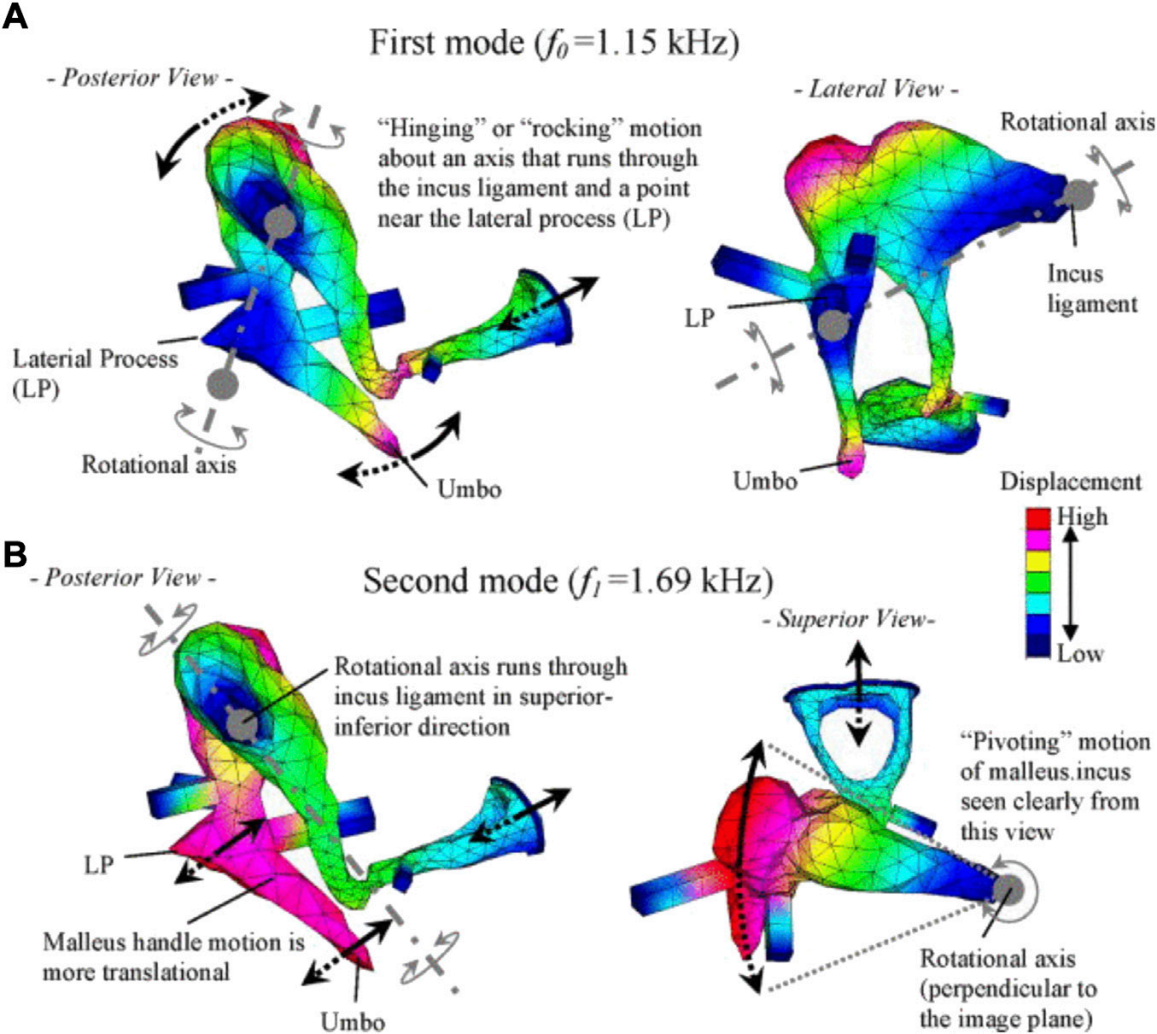
Characteristic ossicular motions described by Homma et al. (2009), reprinted with permission: **(A)** the first mode under air conduction, and **(B)** the second mode under bone conduction.

Reports on FE models of the ossicular chain (Homma et al., 2009) further overcame the difficulty of assessing ossicular motion experimentally. Homma et al. analyzed ossicular resonance modes for bone conduction and air conduction, determining that a “hinging” or “rocking” ossicular motion predominates under air conduction excitation, and the “pivoting” ossicular motion predominates under bone conduction excitation (Homma et al., 2009) (Figure 11).

De Greef et al. (2017) highlighted that the stapes footplate motion, and therefore the middle ear transfer function, is largely influenced by the cochlear impedance, a value that varies among different sources (Merchant et al., 1997; Puria et al., 1997; Aibara et al., 2001; Nakajima et al., 2009). De Greef et al. (2017) also demonstrated that increased damping of the tympanic membrane smoothed the transfer function of the stapes footplate, especially at 3 and 10 kHz. Similarly, O’Connor et al. (2017) modified the tympanic membrane properties showing that 1) at high frequencies, the middle ear pressure gain decreased with increasing tympanic membrane mass, 2) a stiffer tympanic membrane attenuated at low and mid frequencies while boosting high frequencies, and 3) an increase in the shear modulus of the membrane caused an attenuation on low and mid frequencies and boosted high frequencies. Variability of the chosen values in the FE models might explain the discrepancy between experimental and computational results.

Recent models of the tympanic membrane revealed that not just the mass but also the shape of the tympanic membrane plays a role in the sound transmission to the cochlea (Fields et al., 2018) and dictates the force transmitted to the ossicles with the higher force transmitted with cone-shaped tympanic membranes than with flat membranes (Fay et al., 2006).

The effect of bone mass in the ossicular motion has been widely studied with FE models (Nishihara et al., 1993; Gan et al., 2001; Needham et al., 2005; Liu et al., 2016), possibly for its implications in middle ear implantations. Liu et al. (2016) FE models agreed with these experimental studies showing that at high frequencies, the stapes footplate displacement inversely relates to the increasing mass of the incus’ long process (Nishihara et al., 1993; Gan et al., 2001; Needham et al., 2005) and further confirmed that this is also valid for implants placed on the incus’ long process and at the eardrum. Instead, implants placed at the body of the incus decreased stapes footplate displacement at low frequencies. FE models also aid in optimizing implants through the investigation of the effect of diverse materials, shapes, and placement of prostheses on the transfer function of the ossicular chain (Ladak and Funnell, 1996; Ferris and Prendergast, 2000; Kelly et al., 2003; Abel and Lam, 2004).

FE models and experimental analysis powerfully complement and validate each other for the analysis of the middle ear biomechanics. Although they may contain approximations in geometry, material properties, and loading/boundary conditions, mainly because experiments are not yet able to inform us on all of these, FE models can simulate complex systems as middle ear biomechanics and predict behaviors that cannot be analyzed experimentally.

## 8 Unsolved questions on middle ear mechanics

Our understanding of how the middle ear works has developed since the mid-1850s. Fundamental to our current knowledge is the mechanism of impedance matching, which is the role of the outer and middle ear to transfer sound in air to a fluid-filled cochlea. However, much remains yet to be fully understood about the middle ear function and mechanics, and we will consider some of the outstanding questions here.

### 8.1 What is the relative role/contribution of the outer and middle ear in setting the hearing threshold and shaping the audiogram?

It has been debated whether or how much the frequency range and the changes in the threshold at the high and low-frequency ends of the hearing are determined by the middle ear and the contributions of the cochlea. The human audiogram has a band-pass filter shape. At frequencies below 0.5 kHz, thresholds increase with a slope of 12 dB/octave; at frequencies between 0.5 and 2 kHz, thresholds decrease by 6 dB/octave; between 2 and 4 kHz, thresholds are relatively constant; at frequencies above 4 kHz, thresholds increase with a slope of 12 dB/octave; and above 16 kHz the slope is >100 dB/octave.

**FIGURE 12:**
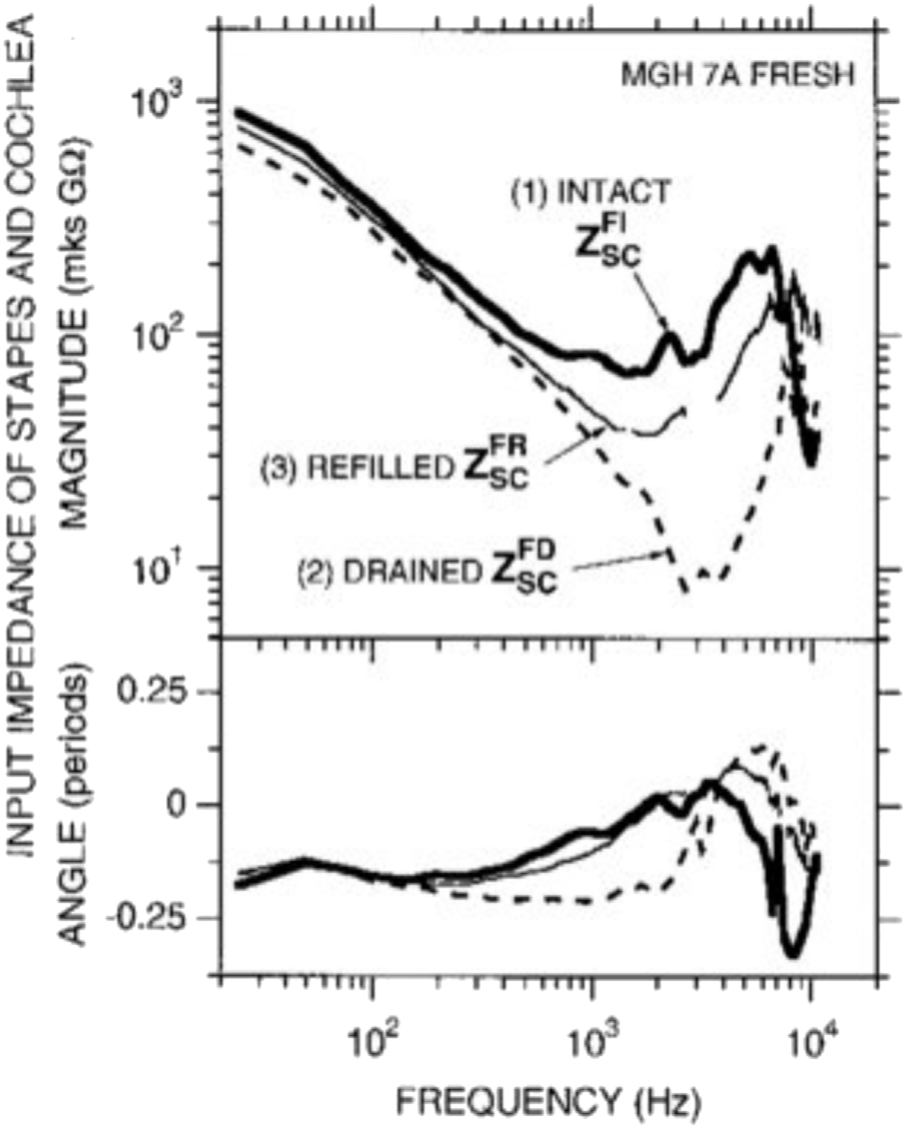
Input impedance of the stapes and cochlea decreases in magnitude and angle with drainage of the cochlea, demonstrating that inner ear fluid plays a crucial role in the transmission of sound. Interestingly, refilling the cochlea allows regaining input impedance angle but not magnitude. Thus cochlear fluid contributes predominantly as a resistive element to the ear input impedance. Reprinted with permission from Ravicz, M. E, et al. (2000).

Cochlear microphonics are electrical signals that can be recorded at the round window and originate from the hair cells. They show a pattern of thresholds similar to the audiogram. Dallos et al. (1971) suggested that the observed pattern is a combination of the middle ear transfer function and basilar membrane vibration at the base of the cochlea. Ravicz and Melcher (2001) demonstrated that the cochlear input impedance is stiffness-controlled below 1 or 2 kHz with an impedance with a -6 dB/octave slope. Interestingly, Aibara et al. (2001) also showed a stiffness-controlled cochlear input impedance but without the expected magnitude increase accompanying an increase in stiffness. Separating the impact of the cochlea input impedance from the middle ear on the audiogram is impossible because one loads the other. Notably, draining the cochlea seems to produce a simple mass-spring system (Figure 12), with the stiffness presumably coming from the annular ligament around the stapes footplate. Thus cochlear fluid contributes predominantly as a resistive element to the ear input impedance.

The audiogram between 4 and 16 kHz suggests a mass-controlled effect given the 12 dB/octave audiogram slope. A mass contribution from the middle ear above 4 kHz was suggested by Withnell and Gowdy (2013), although the data range was limited to 6 kHz in this case. The slope of the audiogram above 16 kHz is too steep to be explained by a mass-controlled system and so does not have a middle ear origin. Instead, the limit must be found in the cochlea due to the limits of the tonotopic cochlear organization (Ruggero and Temchin, 2002). Basically, the high-frequency slope reflects the cochlea’s response above the highest frequency it is tuned to.

### 8.2 How do the vibration patterns of the eardrum and ossicles change at high frequencies?

Laser vibrometry has demonstrated that the eardrum undergoes many local vibrations at high frequencies. These local vibrations are not traveling waves or standing waves. They are vibrations fixed in a location that do not propagate from their site of origin. It is unclear what role these local eardrum vibrations play in audition or why they occur. The conical shape of the eardrum suggests that the output of the sound energy received by the eardrum is the umbo or throat of the conical horn.

Possible modes of ossicle vibration that change with amplitude and frequency have been discussed in great detail by (Hüttenbrink, 1992). The author suggested that sizeable slow pressure changes or mechanical manipulations of the manubrium follow the description provided initially by Helmholtz (1868). For audio frequencies and sound levels that occur during normal conversation, the movement pattern of the middle ear changes: the chain is moving as a fused entity (Hüttenbrink, 1992) around an axis of rotation about the incudomalleolar joint with the axis perpendicular to the direction of stapes motion. Rosowski et al. (2020) posit that the incudomalleolar joint is fixed in space by the anterior malleolar ligament and the posterior incudal ligament, so there is no translational (lateral-medial) motion of this joint associated with this mode. Displacement of the umbo produces rotation at the incudomalleolar joint and displacement of the stapes.

The axis of rotation is no longer as described by Helmholtz or von Békésy but is an “imaginary” axis outside the bodies of the middle ear ossicles. Although not discussed in detail, Figure 8 shows similar results (Rosowski et al., 2020). Their results from the OCT measurements show a lateral-medial whole-body translational component that suggests the incudomalleolar joint is moving in unison without rotation (Figure 8). This would require the synovial joint range of motion to reduce with increasing frequency, so the first mode should decrease frequency-dependent. It further requires that the anchoring of the incudomalleolar joint by the attached ligaments be frequency-dependent, with this mode increasing with increasing frequency. Sim and Puria (2008) suggest that the ligaments act like springs, so their impact on anchoring the incudomalleolar joint would reduce as a function of increasing frequency. They also showed a torsion or twisting of the incudomalleolar joint.

The first mode (Figures 8A,B) is the commonly described piston-like motion of the ossicles, where the motion is in one dimension. A piston-like motion has been inferred from the cochlea input admittance phase, well described by a minimum phase system up to 22 kHz in chinchilla (Ravicz and Rosowski, 2013). The frequency range of hearing for the chinchilla is about 10 octaves, from 60 Hz to 32 kHz (Heffner and Heffner, 1991), so a simple piston-like motion for the stapes describes approximately 9.5 of the 10 octaves. In contrast, the more recent paper by Rosowski et al. (2020), using OCT measurements, suggested that a piston-like motion is dominant up to 8 kHz, with the incudomalleolar joint twisting motion being dominant from 8 to 17 kHz.

As with mode two (Figures 8C,D), this mode requires that the anchoring of the incudomalleolar joint by the attached ligaments is frequency-dependent, with this mode increasing with increasing frequency. A twisting of the incudomalleolar joint is consistent with the rocking motion observed for the stapes footplate at higher frequencies, the stapes footplate having a convex shape.

Complex eardrum vibration patterns and multiple modes of ossicular chain vibration at high frequencies remain to be understood in the context of sound transmission from the ear canal to the cochlea.

### 8.3 How are connective tissue disorders affecting middle ear mechanics?

To date little is known about the effect of disorders of collagenous tissues on the material properties of these tissues in the ear and the mechanics of the middle ear to generate a hearing loss. While modeling and experimental approaches are improving and providing predictive power for the models from a clinical point of view, it is important to understand how genetic and disorder-related predispositions result in middle ear malfunction and what measures can be taken to restore normal function. Developing new treatments for patients with diseases that affect bone formation and also result in hearing loss, such as Paget’s disease or osteogenesis imperfecta (OI), may be relevant also for the general population. For people with brittle bones, conventional treatments for hearing loss do not offer a reliable solution (Ugarteburu et al., Forthcoming 2022). However, a detailed understanding of the ear structure and function in these conditions is essential for developing new therapies. Little data exist on the material properties of the ligaments and bones, and their effect on middle ear mechanics. The majority of these are also on mice models of the disease, such as those for OI (Bonadio et al., 1990; Altschuler et al., 1991; Chen et al., 2007; Stankovic et al., 2007; Pokidysheva et al., 2013; De Paolis et al., 2021; Patel et al., 2022). Studies from small animals must then be translated to humans to be clinically relevant. Thus further studies that aim to understand the differences and similarities between these two hearing systems are needed.

## 9 Conclusion

The middle ear is important for hearing and matches the impedance between the air and the fluid-filled cochlea allowing the acoustic energy to reach the inner ear, where it is transformed into action potentials that the brain can interpret as speech, music, or noise. The early workings were well described in the 18th century, and more sophisticated measurement methods have refined our understanding of tympanic membrane and middle ear ossicle movements. While modeling and experimental approaches are improving, questions still need to be answered to fully understand the middle ear mechanics and the hearing loss associated with its malfunctioning.

## 10 Appendix: Methods to measure middle ear function

**FIGURE 13:**
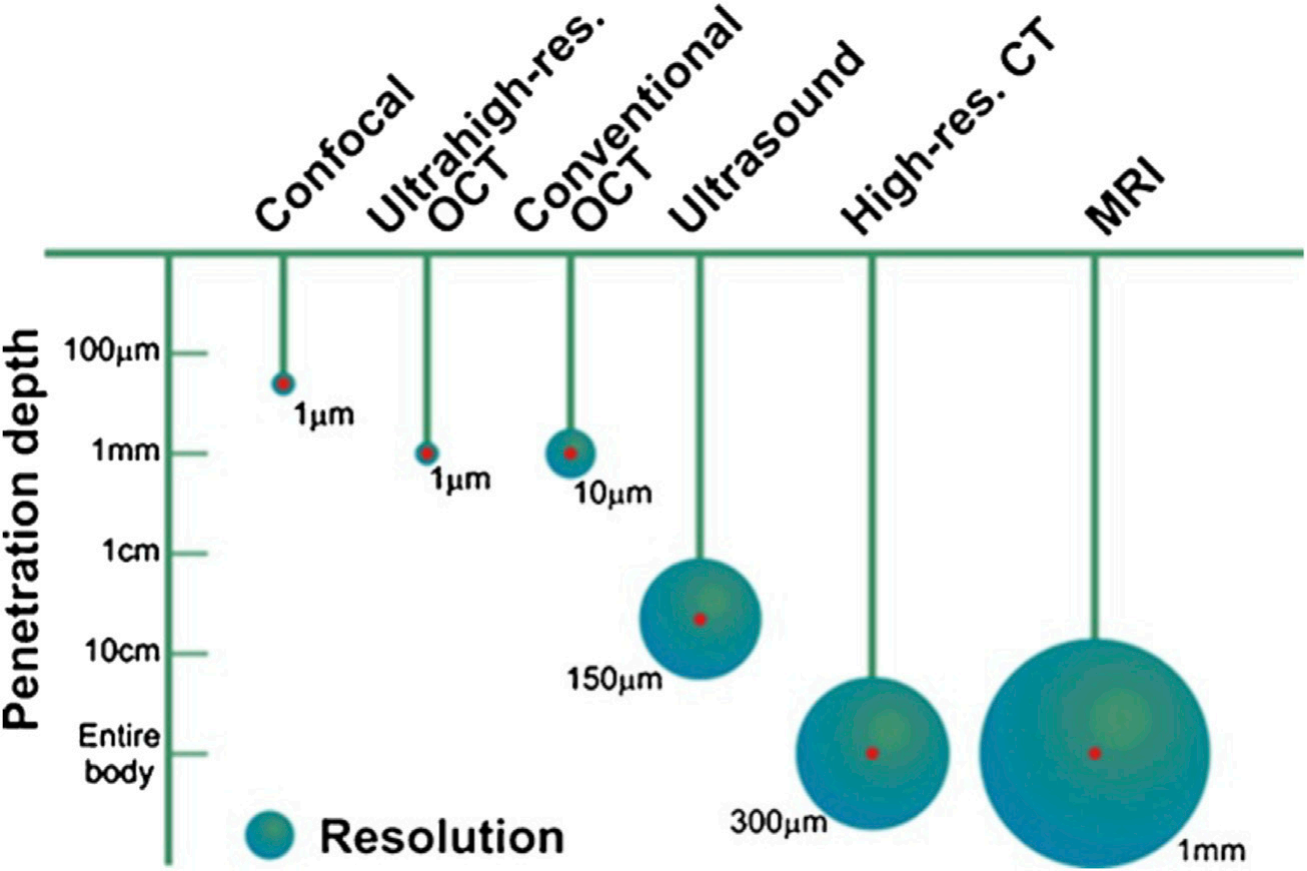
Penetration depth and resolution of different imaging modalities, reprinted with permission from (Popescu et al., 2011).

Vibration amplitudes of the middle ear ossicles are extremely small. The first methods to visualize such vibrations included stroboscopic illumination and visual examination. It is understandable that initially, only responses to non-physiological inputs were measured. With the development of novel, more sensitive methods, it is now possible to measure vibrations at physiological sound levels in the ear. Figure 13 shows currently available methods to measure middle ear mechanics.

### 10.1 Stroboscopic methods

The stroboscopy was the first method to quantify vibration patterns of key structures in the cochlea, such as the basilar membrane. It is an imaging technique where the target structure is imaged continuously while illuminated by intermittent light, the frequency of the intermittent light close to the frequency of movement of the object under investigation. The technique is not spatially selective, and large vibrations of the target structures are required for a response. For example, for studying the vibration patterns of the inner ear soft tissue structures, von Békésy delivered sound levels well above 100 dB SPL (sound level re 20 µPa).

### 10.2 The Mössbauer method

A radioactive source is placed on the target structure. A detector monitors the emitted radiation. Counts are locked to the acoustic signal, and period histograms with the sufficient resolution are constructed from the recordings. Velocity estimates are fitted to the recorded data. (Rhode, 1974; Tonndorf, 1977; Gundersen et al., 1978; Kringlebotn et al., 1979; Gummer et al., 1989; Ruggero and Rich, 1991).

### 10.3 Capacitive probe measurements

Von Békésy used the capacitive probe to measure the middle ear mechanics and map the tympanic membrane’s vibration pattern. Capacitive probes require the probe to be placed in the proximity of the object of interest, the stapes footplate. Therefore, this needs to access the footplate through the inner ear, thus removing the fluid from the cochlea and consequently removing the load that the cochlea applies to the middle ear (Vlaming and Feenstra, 1986).

### 10.4 Laser doppler velocimetry

The interaction of two coherent laser beams, a reference and a target beam, is used to calculate the movement of the beams oriented towards the target structure. Displacements less than 10^–8^ m at frequencies between 100 Hz and 100 kHz can be measured. The measurements are point measurements. To evaluate areas, multiple measurements at the selected location must be conducted (Drain, 1980; Nuttall et al., 1991; Stasche et al., 1993; Ulfendahl et al., 1996; Maier et al., 2013; Masalski et al., 2021).

### 10.5 Holography

Holography is a technique that uses the pattern of interference between highly coherent beams determined by the reflection or transmission of the light beams through a target structure to reconstruct the amplitude and phase distribution of a coherent wave. Two beams are required for two-dimensional (2D) and three for three-dimensional (3D) holograms (Powell and Stetson, 1965; Khaleghi et al., 2013; Khaleghi et al., 2015a; Khaleghi et al., 2015b). For moving target structures, the resulting fringes can be used to simultaneously determine the selected sites’ vibrations.

### 10.6 Speckle contrast imaging

When coherent light interacts with a random scattering medium, the scattered light will interfere constructively and destructively, resulting in a pattern of bright spots or speckles. This pattern is monitored with a camera over time. Movements of the scattering medium lead to fluctuations of the speckles and subsequent intensity variations of a detector monitoring the speckles. Temporal and spatial speckle statistics provide information on the movement of the scattering medium (Yamaguchi et al., 1990; Wada et al., 2002).

### 10.7 Optical coherence tomography

Optical Coherence Tomography (OCT) is an optical, non-invasive imaging technique. In its simplest form, the emitted radiation from a single low-coherent light source, typically near-infrared, is split into a reference and a target beam. The light in the reference beam reflected by a mirror and the light backscattered from the tissue in the target beam are combined in a coupler and recorded by a single-point detector. The recordings allow a reconstruction of the target structures. Hereby, the photon count at the detector and the quality of the detector determines the image quality. While the penetration depth of the light in tissue is relatively short, 1 mm, a decent spatial resolution of about 1–10 µm can be achieved (Pitris et al., 2001; Popescu et al., 2011; Subhash et al., 2012; Chang et al., 2013; Macdougall et al., 2015; Jeon et al., 2019a; Burwood et al., 2019; Oh et al., 2020).
